# Safety of microneurosurgical interventions for superficial and deep-seated brain metastases: single-center cohort study of 637 consecutive cases

**DOI:** 10.1007/s11060-023-04478-1

**Published:** 2023-11-10

**Authors:** Stefanos Voglis, Luis Padevit, Christiaan Hendrik Bas van Niftrik, Vincens Kälin, Benjamin Beyersdorf, Raffaele Da Mutten, Vittorio Stumpo, Jacopo Bellomo, Johannes Sarnthein, Victor Egon Staartjes, Alessandro Carretta, Niklaus Krayenbühl, Luca Regli, Carlo Serra

**Affiliations:** 1 Department of Neurosurgery, Clinical Neuroscience Center, University Hospital and University of Zurich, Rämistrasse 100, 8091 Zurich, Switzerland; 2 Department of Biomedical and NeuroMotor Sciences (DIBINEM), University of Bologna, Bologna, Italy

**Keywords:** Brain metastases, Adverse events, Complications, Surgical resection

## Abstract

**Purpose:**

Microneurosurgical techniques have greatly improved over the past years due to the introduction of new technology and surgical concepts. To reevaluate the role of micro-neurosurgery in brain metastases (BM) resection in the era of new systemic and local treatment options, its safety profile needs to be reassessed. The aim of this study was to analyze the rate of adverse events (AEs) according to a systematic, comprehensive and reliably reproducible grading system after microneurosurgical BM resection in a large and modern microneurosurgical series with special emphasis on anatomical location.

**Methods:**

Prospectively collected cases of BM resection between 2013 and 2022 were retrospectively analyzed. Number of AEs, defined as any deviations from the expected postoperative course according to Clavien–Dindo-Grade (CDG) were evaluated. Patient, surgical, and lesion characteristics, including exact anatomic tumor locations, were analyzed using uni- and multivariate logistic regression and survival analysis to identify predictive factors for AEs.

**Results:**

We identified 664 eligible patients with lung cancer being the most common primary tumor (44%), followed by melanoma (25%) and breast cancer (11%). 29 patients (4%) underwent biopsy only whereas BM were resected in 637 (96%) of cases. The overall rate of AEs was 8% at discharge. However, severe AEs (≥ CDG 3a; requiring surgical intervention under local/general anesthesia or ICU treatment) occurred in only 1.9% (n = 12) of cases with a perioperative mortality of 0.6% (n = 4). Infratentorial tumor location (OR 5.46, 95% 2.31–13.8, p = .001), reoperation (OR 2.31, 95% 1.07–4.81, p = .033) and central region tumor location (OR 3.03, 95% 1.03–8.60) showed to be significant predictors in a multivariate analysis for major AEs (CDG ≥ 2 or new neurological deficits). Neither deep supratentorial nor central region tumors were associated with more major AEs compared to convexity lesions.

**Conclusions:**

Modern microneurosurgical resection can be considered an excellent option in the management of BM in terms of safety, as the overall rate of major AEs are very rare even in eloquent and deep-seated lesions.

**Supplementary Information:**

The online version contains supplementary material available at 10.1007/s11060-023-04478-1.

## Introduction

Brain metastases (BM) are the most common form of malignant central nervous system tumors, and their incidence is increasing due to improved systemic therapies and therefore survival rates of patients with solitary tumors. In recent years new treatment modalities have arisen mostly incorporating targeted and immunotherapeutic agents [1] and yet microneurosurgical resection with adjuvant radiotherapy has remained the mainstay for local control, especially for patients with solitary BM [2, 3]. Traditionally, microneurosurgical resection of BM has been considered primarily for solitary or symptomatic lesions, or in patients with unknown primary tumor for histopathological diagnosis. Multiple and/or asymptomatic metastasis, or those in deep or so-called “eloquent” regions are preferentially referred to radiotherapy, either in single or in multiple sessions [2]. However, the introduction of new therapeutic modalities and concepts [4–6] together with continuously improving microneurosurgical techniques warrants the need to reassess the value of surgical tumor burden reduction within multimodal treatment concepts [7], and the safety of microneurosurgical BM resection not only in solitary and superficial lesions but also in multiple, highly eloquent and deep-seated tumors.

Adverse events (AE), defined as any deviation from the expected postoperative course, can significantly affect patients’ outcome due to the delay of adjuvant or subsequent treatment with a possible negative effect on overall survival [8, 9]. Historically, the rate of AEs after radiotherapy, particularly radiosurgery, is assumed to be lower [10, 11] than after micro-neurosurgery [12–14], but modern and large series with a clear definition and systematic reliably reproducible grading of AEs are scarce, particularly with respect to different anatomical BM locations.

With the introduction of new treatment modalities for BM, it is crucial to reassess the safety of modern microneurosurgical BM resection especially considering multiple, central region and deep-seated lesions. Here we report a large series of 666 patients of which 537 underwent microneurosurgical resection in the last decade for histologically confirmed BM. Particular emphasis is put on surgical outcome and standardized assessment of AEs characteristics.

## Methods

### Study cohort, data acquisition and ethical considerations

All patients who underwent microneurosurgical resection or biopsy of histologically confirmed BM between January 2012 and June 2022 at our institution were included. Patient records were extracted from our prospectively recorded institutional registry [15]. The registry was approved upfront by the local ethics review board (PB-2017-00093) and internationally registered at clinicaltrials.gov (NCT01628406). Parameters extracted from the registry contained age, sex, American Society of Anesthesiologists (ASA) risk classification, length of stay (LOS), modified Rankin scale (mRS), Karnofsky Performance Scale (KPS), surgical characteristics and AEs at discharge. mRS and KPS were used as general clinical performance scales. Complications are defined as any deviation of the usual, expected postoperative course and are graded according to the Clavien–Dindo Grading system (CDG, see Online resource 1) [16–18]. For ease of handling, for each case only the complication with the highest CDG entered further analysis, if not stated otherwise.

All data were collected by staff neurosurgeons at the time of hospital admission, surgery, hospital discharge, and at each outpatient follow-up visit. Discharge reports are validated by the attending neurosurgeon responsible for the patient. In addition, all AEs are validated at the monthly department meeting and at the monthly morbidity and mortality meeting to ensure an accurate data collection. Data entry is performed by neurosurgeons only, and each new team member is provided with introductory training and written instructions in the form of a standardized operating procedure and is required to obtain a certificate on the correct use of the clinical and complication scores.

For each case, the number of craniotomies, surgical modalities such as intraoperative ultrasound, neuromonitoring and intraoperative magnetic resonance imaging (MRI), as well as number of metastases and anatomic gyral location of resected tumors were additionally extracted from the hospital’s electronic medical record system. Pre- and postoperative T1-weighted MRI images with gadolinium contrast were used to determine anatomical location of the BM and were confirmed by neuroradiological reports and an experienced consultant neurosurgeon. Tumor location was divided into infra- and supratentorial and further stratified into superficial and deep-seated. Deep lesions were defined as tumor location in the cuneus, precuneus, corona radiata, basal ganglia, thalamus, cingulate gyrus, ventricles, operculum, medial and lateral occipitotemporal gyrus, orbital gyrus, insula, clivus, parahippocampal gyrus, corpus callosum, pineal region, gyrus rectus, medulla oblongata, pons as depicted in Online resource 2.

### Statistical analysis

All data processing and analysis steps were performed with R Studio (Version 1.4, R Studio Inc.) [19] using open-source libraries. Plotting of anatomical locations on a reference brain atlas was done using the *coldcuts* R package [20]. Missing values were considered missing at random and therefore omitted from all analysis. Continuous variables are given as means and standard deviation (SD) whereas categorical variables are reported as numbers and percentages of total. Uni- and multivariate logistic regression analysis was performed to find predictive features for AEs. The statistical tests used are additionally indicated in the figure captions or the main text. P-values < 0.05 were considered statistically significant. Further study cohort stratification was based on the occurrence of major AEs at discharge which are defined as the occurrence of any AE with CDG ≥ 2 or a new neurological deficit at discharge.

### Data and script availability

Raw data and analysis scripts are available from the corresponding author upon reasonable request.

## Results

### Study cohort characteristics

A total of 664 patients which underwent surgery of BM were included in the study cohort. Sex was roughly equally distributed with 51% male (n = 337) and 49% female (n = 327). Mean patient age was 61 years and the BM most frequently originated from the lung (44%, n = 294) followed by melanoma (25%, n = 163) and breast cancer (11%, n = 74) as depicted in Table 1. In most patients a singular BM was present (50%, n = 336), whereas 2–4 lesions were present in 33% and > 10 lesions in 8.6% of the patients (see Table 1).

**Table 1:** Patient cohort and surgical characteristics

Characteristic	N = 664
Sex	
Male	337 (51%)
Female	327 (49%)
Age	61.04 (12.47)
Primary tumor	
Lung	294 (44%)
Melanoma	163 (25%)
Breast	74 (11%)
Gastrointestinal	45 (6.8%)
Renal	21 (3.2%)
Head and neck	17 (2.6%)
Urological	17 (2.6%)
Gynecological	13 (2.0%)
Unknown	9 (1.4%)
Sarcoma	6 (0.9%)
Other	5 (0.8%)
Number of metastases	
1	334 (50%)
2–4	218 (33%)
5–10	55 (8.3%)
> 10	57 (8.6%)
Intraoperative ultrasound	
Yes	520 (78%)
No	144 (22%)
Intraoperative MRI	
No	637 (96%)
Yes	27 (4.1%)
Intraoperative navigation	
Yes	528 (89%)
No	65 (11%)
Unknown	71
Intraoperative neuromonitoring	
No	539 (81%)
Yes	124 (19%)

Statistics presented: mean (± SD); n (%)

*MRI*   magnetic resonance imaging

### BM and surgical characteristics

The most frequently used intraoperative tools were intraoperative neuronavigation (89%) and intraoperative ultrasound (78%). Not surprisingly, neuromonitoring (19%) and intraoperative MRI (4%) were used less often as shown in Table 1. Most of the surgeries were resections (96%) via one craniotomy (89%), while 6% (n = 40) of the patients underwent two and four patients underwent three craniotomies during the same surgery. Biopsy only cases accounted for 4% (n = 27) and 19% of cases were reoperations with previous BM resections (see Table 2, Overall).

**Table 2:** Lesion and surgery characteristics

Characteristic	Overall, N = 664	Any major AE at discharge		p-value
		No N = 614 (92.5%)	Yes N = 50 (7.5%)	
Central region				0.6
No	588 (89%)	545 (89%)	43 (86%)	
Yes	76 (11%)	69 (11%)	7 (14%)	
Infratentorial location				** < 0.001**
No	521 (78%)	489 (80%)	30 (60%)	
Yes	145 (22%)	125 (20%)	20 (40%)	
Supratentorial lesions				0.2
Superficial	352 (71%)	336 (71%)	16 (59%)	
Deep-seated	145 (29%)	134 (29%)	11 (41%)	
Sidedness				**0.013**
Right	329 (50%)	309 (50%)	20 (40%)	
Left	290 (44%)	269 (44%)	21 (42%)	
Both	28 (4.2%)	23 (3.7%)	5 (10%)	
Midline	17 (2.6%)	13 (2.1%)	4 (8.0%)	
Type of surgery				0.7
Resection	637 (96%)	588 (96%)	49 (98%)	
Biopsy	27 (4.1%)	26 (4.2%)	1 (2.0%)	
Primary surgery				0.2
Yes	534 (81%)	498 (82%)	36 (73%)	
No	126 (19%)	113 (18%)	13 (27%)	
Unknown	4	3	1	
No. of craniotomies				0.2
1	593 (89%)	550 (90%)	43 (86%)	
2	40 (6.0%)	35 (5.7%)	5 (10%)	
3	4 (0.6%)	3 (0.5%)	1 (2.0%)	
Biopsy	27 (4.1%)	26 (4.2%)	1 (2.0%)	
ASA risk classification				0.3
1	10 (1.5%)	10 (1.7%)	0 (0%)	
2	206 (32%)	194 (32%)	12 (24%)	
3	377 (58%)	347 (58%)	30 (60%)	
4	58 (8.9%)	50 (8.3%)	8 (16%)	
5	1 (0.2%)	1 (0.2%)	0 (0%)	
Unknown	12	12	0	
Urgency of the operation				> 0.9
Elective	524 (88%)	480 (88%)	44 (88%)	
Emergency	69 (12%)	63 (12%)	6 (12%)	
Unknown	71	71	0	

Major AE at discharge were considered as CDG ≥   2 or new neurological deficits. Statistics presented: n (%). Pearsons Chi-squared test (for all n ≥ 5) and Fisher’s exact test (for all n < 5). Statistically significant p-values are marked in bold

*AE* adverse event, *ASA* American Society of Anesthesiologists

**Fig. 1:**
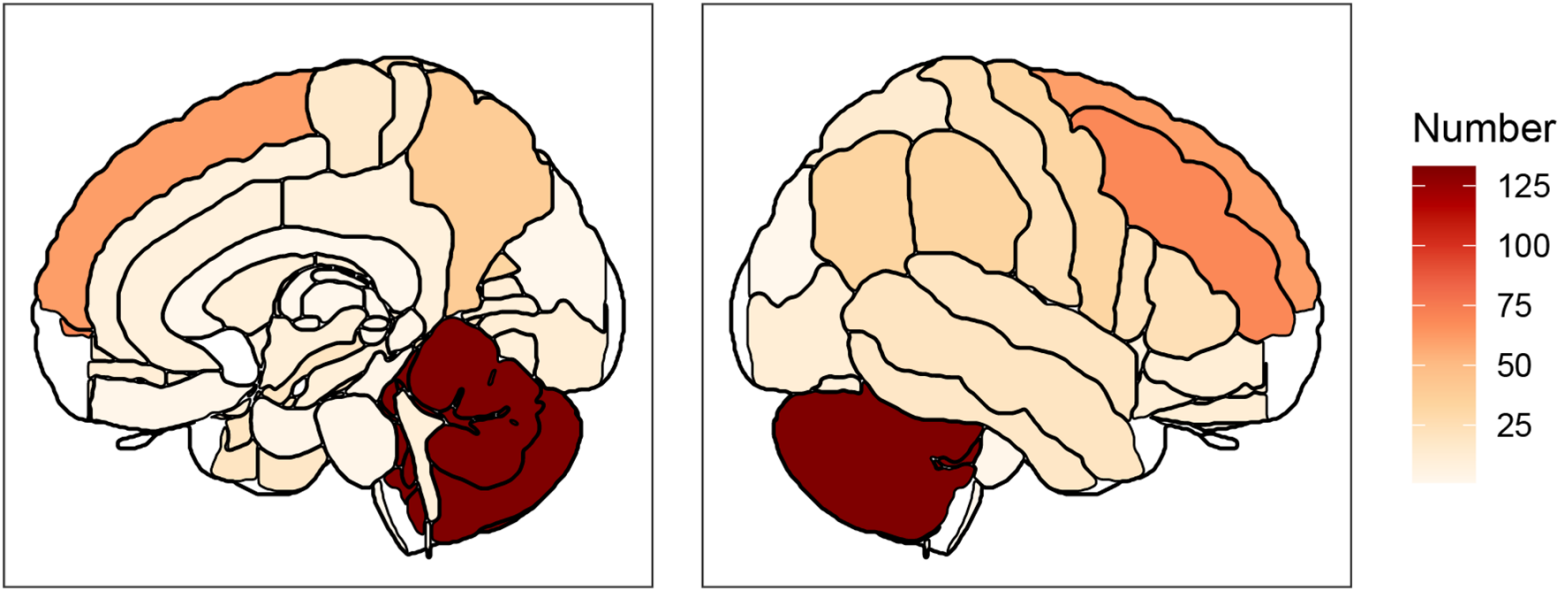
Anatomical BM location. *BM* brain metastases

78% of cases were located supratentorial, whereas 22% were located infratentorial (n = 145, see Table 2, Overall). Of the supratentorial lesions, 71% had a convexity and 29% a subcortical/deep location. 11% (n = 76) BM were located in the central region, which is comprised of the pre-, post- para- and subcentral gyrus [21]. Online resource 2 and Fig. 1 illustrate the gyral localization of the resected BM in the study cohort. Roughly half of the lesions were either located on the right or left side (50% vs. 44% respectively) while the minority were in the midline (2.6%, n = 17).

### Frequency of adverse events and clinical outcomes

The occurrence of any AE at discharge was recorded in 8% (n = 53, see Online resource 3) of the cases, most of which were recorded as CDG 1 and 2 (see Online resource 3), meaning that these AEs did not require any invasive interventions, but only pharmacological or no treatment. Severe AE requiring invasive interventions (CDG 3) or ICU stay (CDG 4) were present in only 1.9% (n = 12) and the mortality rate was 0.6% (n = 4, CDG 5). New neurological deficits occurred in 2% of the entire study cohort (n = 13, see Online resource 3), followed by urinary tract infections, pulmonary artery embolism (each 0.8%, n = 5), postoperative hemorrhage and pneumonia (each 0.6%, n = 4).

**Fig. 2:**
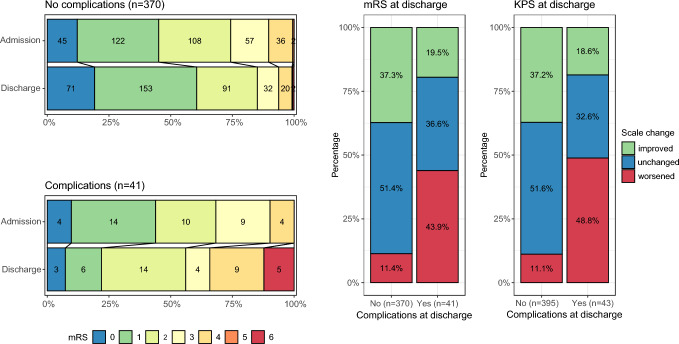
Clinical outcome scale changes at discharge. Percentages of mRS scores at admission (upper row) and discharge (lower row) for patients without AE (**A**) and for patients with AE (**B**). Changes of mRS (**C**) and KPS (**D**) at discharge relative to admission stratified for the occurrence of AE. AE at discharge were considered as CDG ≥ 2 or new neurological deficits. *AE* adverse event, *mRS* modified Rankin Scale, *KPS *Karnofsky Performance Status

Stratifying the study cohort according to the occurrence of major AEs (defined as CDG ≥ 2 or new neurological deficits at discharge), Table 2 shows the occurrence of major AEs for the different anatomic and surgical parameters. Cases with major AEs were more frequently located infratentorial (40% vs. 20%, p ≤ 001, Pearson’s Chi-squared test) or in the midline (8% vs. 2%, p = 0.013, Fisher’s exact test; see Table 2). Major AEs were not associated with deep-seated lesions, the number of craniotomies or emergency operations (see Table 2). Figure 2 shows the postoperative change in clinical outcome scales for the two groups: Patients who experienced an AE had higher mRS scales at discharge compared to admission (see Fig. 2B) compared to patients who did not experience any AE (see Fig. 2A). In addition, considering the relative changes in mRS (see Fig. 2C) and KPS (see Fig. 2D) between discharge and admission, more patients with an AE suffered from a worsening of mRS (44%) and KPS (49%) compared to patients without AEs (11% each, p < 0.001 for mRS and KPS, Pearson’s chi-squared test).

### Logistic regression and survival analysis

In univariate logistic regression analysis, ASA status (OR 1.6, 95% CI 1.01–2.54, p = 0.044), tumor location in the midline (OR 3.94, 95% CI 1.04–12.3, p = 0.037) and cerebellar tumor location (OR 3.44, 95% CI 1.73–6.95, p = 0.005) were significant predictors of postsurgical major AEs (see Online resource 4). Furthermore, in a subsequent multivariate logistic regression analysis, cerebellar BM location (OR 5.46, 95% 2.31–13.8, p = 0.001), reoperation (OR 2.31, 95% 1.07–4.81, p = 0.033, see Table 3) and central region tumors (OR 3.03, 95% 1.03–8.60) were all associated with the occurrence of postoperative AEs. Looking at overall survival (OS), Kaplan–Meier curve analysis showed significant differences in OS between patients with major AEs and the ones without AEs (p = 0.044, log-rank test, see Online resource 5). Additionally, patients with higher BM load (n ≥ 5 BM) showed significantly impaired OS rates compared to patients with fewer BM (n < 5; p < 0.001 log-rank test, see Online resource 6).

**Table 3:** Multivariate logistic regression model of major AE at discharge

Characteristic	OR	95% CI	p-value
Age	1.00	0.98, 1.03	0.75
Sex			0.42
Female	–	–	
Male	1.29	0.70, 2.41	
ASA risk classification	1.43	0.86, 2.42	0.17
Primary surgery			**0.033**
Yes	–	–	
No	2.31	1.07, 4.81	
Number of craniotomies			0.72
1	–	–	
> 1	1.42	0.35, 4.73	
Biopsy	0.55	0.03, 3.13	
Central region			**0.045**
No	–	–	
Yes	3.03	1.03, 8.60	
Sidedness			0.30
Left	–	–	
Midline	2.44	0.58, 8.75	
Right	0.73	0.37, 1.43	
Both	1.44	0.31, 5.93	
Neocortical location			**0.001**
Convexity	–	–	
Deep	2.38	0.93, 6.23	
Cerebellar	5.46	2.31, 13.8	
Extraaxial	2.90	0.41, 12.8	
Urgency of the operation			0.97
Elective	–	–	
Emergency	1.02	0.36, 2.48	

Major AE at discharge were considered as CDG  ≥  2 or new neurological deficits. Statistically significant p-values are marked in bold

*AE*  adverse event, *OR*  odds ratio, *CI*   confidence interval, *ASA*   American Society of Anesthesiologists

## Discussion

The treatment of patients with metastatic cancer is rapidly changing due to the introduction of novel therapeutic modalities, most notably targeted and immunotherapeutic systemic agents [1]. This resulted in an improved overall survival in these patients and an increasing incidence of the development of BM [4–6]. However, due to the changing multimodal treatment regiments in BM patients, the role and safety of microneurosurgical BM resection needs to be reassessed in the light of other treatment modalities. Technological development has advanced modern micro-neurosurgery forward with numerous technical developments to maximize intraoperative patient safety and improve the extent of resection [22–26]. This is reflected in the low overall rate of AE in our patient cohort with only few AEs considered as severe based on the CDG grading and without any difference between cortical, central or deep-seated lesions. Our data shows that modern microneurosurgical resection can be considered as safe also in the case of deep located or central region lesions. Infratentorial BM or non-primary surgical resections both were independent predictors of postsurgical major AEs, which has also been described in the literature before [12, 27], whereas central region location did not reach statistical significance in our cohort. Extra care in perioperative management must be applied in these situations to avoid AEs.

Patients with AEs showed decreased overall survival in our Kaplan–Meier analysis, which might be due to the subsequent delay of postoperative therapies (which is already known from glioma surgery [8]) with a corresponding effect on overall survival. Furthermore, patients with AEs did show worse functional outcome as measured by the mRS and KPS scales. Since both overall survival and functional outcome decreased after AEs, the importance of perioperative safety must be emphasized.

The reported rates of AEs might appear at first sight similar to previous reports [12, 14]. However, on the one hand modern case series are scarce [14, 28, 29], on the other hand AEs are often reported in a non-standardized or non-reproducible manner, or focused primarily on “neurosurgical relevant” AEs [13, 30] leading to a possible underestimation of total number of AEs. We adopt a broad definition of AEs as any deviation from the normal postoperative course. As suggested in the literature [4], this methodological approach is more rigorous in assuring that no AE is missed.

In general, there seems to be a continuing trend of decreasing incidence of AEs, presumably due to newly introduced technical nuances that increase operative safety. If in the ‘80s reported AE could be as high as 27% [28, 30, 31], more recent series report a AE rate of 10–12% [12], Our results, although they originate from a prospective registry (which notoriously leads to much higher reported AE rate [32]) and rely on a broader definition of AE, seem to suggest an even lower AE rate. As such, based on our data, modern microneurosurgical resection can be considered as safe in terms of the overall AE rate and severity. Recent studies of radiosurgical AEs reported an overall AE rate similar to our findings with 2.9% [11], 7% [10] and 6.6% [33] respectively. Interestingly, the definition of AEs was rather narrow and included mostly the occurrence of new neurological deficits only, without considering other forms of AEs as it is the case of the CDG grading. Still, our data suggest that in the current era, thanks to continuous improvement of microneurosurgical technique, the overall rate of AEs after microneurosurgical BM resection might be comparable to that after radiotherapy both in incidence and in severity, although our study was not designed to specifically address this issue.

In our study, anatomical infratentorial tumor location as well as reoperation were shown to be associated with postoperative AEs, which has been described previously [12, 27]. Particularly interesting appears to be the fact that the rate of AEs for supratentorial lesions did not seem to significantly differ between superficial, central and deep-seated BM. This confirms the role of microneurosurgical resection techniques [21, 34] and supports that microneurosurgical resection can be safely performed also in the case of central as well as deep-seated lesions.

We are completely aware of possible limitations of this study, which consists of its retrospective as well as single-center design and the inter-surgeon variability regarding the anatomical localization of the lesions. However, regarding the last point, each anatomical localization was confirmed by the authors of this study and thus bias should be minimized. Taken together, the AE rates presented in this study should be compared to other centers to confirm the overall low incidence of severe AE following microsurgical resection of deep or superficially seated BM.

## Conclusions

Microneurosurgical resection is an excellent option in modern treatment of BM in terms of safety. Incidence of any AE, particularly severe ones, is low even in multiple, central and deep-seated lesions. Infratentorial tumor location and reoperation are associated with postoperative AEs and should warrant a closer perioperative patient monitoring.

### Supplementary Information

Below is the link to the electronic supplementary material.

Supplementary file1 (DOCX 277 kb)

## Data Availability

The datasets generated during and/or analysed during the current study are available from the corresponding author on reasonable request.

## Abbreviations

AE: Adverse event
ASA: American Society of Anesthesiologists
BM: Brain metastases
CDG: Clavien–Dindo-Grade
ICU: Intensive care unit
KP: Karnofsky performance status
MRI: Magnetic resonance imaging
mRS: Modified Rankin Scale
OR: Odds ratio
OS: Overall survival
SD: Standard deviation
