# Supplementary material for: Safety of microneurosurgical interventions for superficial and deep-seated brain metastases: single-center cohort study of 637 consecutive cases
Source: J Neurooncol. 2023 Nov 10;165(2):271–8. doi: 10.1007/s11060-023-04478-1 (PMC10689541; doi:10.1007/s11060-023-04478-1)
Supplement: Supplementary file 1 — Supplementary file1 (DOCX 277 kb) [file 11060_2023_4478_MOESM1_ESM.docx]

## Original article Research:

## Safety of Microneurosurgical Interventions for Superficial and Deep-Seated Brain Metastases: Single-Center Cohort Study of 637 Consecutive Cases

Stefanos Voglis (ORCID 0000-0002-1514-1442)^1,†,*^, Luis Padevit (ORCID [0000-0003-2461-0404](https://orcid.org/0000-0003-2461-0404))^1,†^, Christiaan Hendrik Bas van Niftrik (ORCID 0000-0003-0930-8717)^1^, Vincens Kälin^1^, Benjamin Beyersdorf (ORCID 0000-0001-9272-3115)^1^, Raffaele Da Mutten (ORCID 0000-0002-2645-0865)^1^, Vittorio Stumpo (ORCID 0000-0002-8175-0035)^1^, Jacopo Bellomo (ORCID 0009-0005-8945-4838)^1^, Johannes Sarnthein (ORCID 0000-0001-9141-381X)^1^, Victor Egon Staartjes (ORCID 0000-0003-1039-2098)^1^, Alessandro Carretta, MD^1,2^, Niklaus Krayenbühl (ORCID 0000-0002-4973-7527)^1^, Luca Regli (ORCID 0000-0003-4639-4474)^1^, Carlo Serra (ORCID 0000-0002-7305-550X)^1^

^1^Department of Neurosurgery, Clinical Neuroscience Center, University Hospital and University of Zurich, Zurich, Switzerland

^2^Department of Biomedical and NeuroMotor Sciences (DIBINEM), University of Bologna, Bologna, Italy

Running title: Reassessing the safety of modern microneurosurgical brain metastases resection

†These authors contributed equally.

***Correspondence:**

Stefanos Voglis MD – [stefanos.voglis@usz.ch](mailto:stefanos.voglis@usz.ch)

ORCID: 0000-0002-1514-1442

Department of Neurosurgery, Clinical Neuroscience Center

University Hospital Zurich

Frauenklinikstrasse 10, 8091 Zurich, Switzerland

**Word count**

Total: 3517

Abstract: 318

Text: 2335

Reference: 864

| **Grade** | **Definition** |
| --- | --- |
| **1** | Any deviation from the normal postoperative course without the need for pharmacological treatment or surgical, endoscopic, and radiological interventions. Allowed therapeutic regimens are drugs as antiemetics, antipyretics, analgetics, diuretics, electrolytes, and physiotherapy. This grade also includes wound infections opened at the bedside. |
| **2** | Requiring pharmacological treatment with drugs other than such allowed for grade I complications. Blood transfusions and total parenteral nutrition are also included. |
| **3** | Requiring surgical, endoscopic, or radiological intervention. |
| **3a** | Intervention not under general anesthesia. |
| **3b** | Intervention under general anesthesia. |
| **4** | Life-threatening complication requiring intensive care unit stay |
| **4a** | Single-organ dysfunction (including dialysis). |
| **4b** | Multiorgan dysfunction. |
| **5** | Death of a patient. |

**Online resource 1 Clavien-Dindo Grading System (CDG)**

| **Supratentorial: Superficial** | | **Supratentorial: Deep** | | **Infratentorial** | |
| --- | --- | --- | --- | --- | --- |
| Total number | N = 352 (55%) | Total number | N = 142 (22%) | Total number | N = 145 (23%) |
| Middle frontal gyrus | 68 (19%) | Cuneus | 40 (28%) | Cerebellar | 142 (98%) |
| Superior frontal gyrus | 59 (17%) | Precuneus | 26 (18%) | Deep | 3 (2.1%) |
| Inferior parietal lobule | 32 (9.1%) | Orbital gyrus | 11 (7.7%) | Pons | 2 |
| Precentral gyrus | 30 (8.5%) | Parahippocampal gyrus | 10 (7%) | Medulla oblongata | 1 |
| Postcentral gyrus | 24 (6.8%) | Fusiform gyrus | 9 (6.3%) |  |  |
| Inferior frontal gyrus | 23 (6.5%) | Ventricular | 9 (6.3%) |  |  |
| Superior temporal gyrus | 20 (5.7%) | Insula | 8 (5.6%) |  |  |
| Middle temporal gyrus | 19 (5.4%) | Cingulate gyrus | 8 (5.6%) |  |  |
| Paracentral lobule | 17 (4.8%) | Operculum | 5 (3.5%) |  |  |
| Inferior temporal gyrus | 17 (4.8%) | Basal ganglia | 4 (2.8%) |  |  |
| Superior parietal lobule | 14 (4%) | Corona radiata | 4 (2.8%) |  |  |
| Middle occipital gyrus | 13 (3.7%) | Pineal gland | 2 (1.4%) |  |  |
| Inferior occipital gyrus | 10 (2.8%) | Gyrus rectus | 2 (1.4%) |  |  |
| Subcentral gyrus | 5 (1.4%) | Corpus callosum | 1 (0.7%) |  |  |
| Superior occipital gyrus | 1 (0.3%) |  |  |  |  |

**Online resource 2** **Tumor locations stratified into supra- and infratentorial location as well as gyral anatomy.** Statistics presented: n (%), N = number

| **Characteristic** | **N = 664** |
| --- | --- |
| **AE at discharge** |  |
| No | 611 (92%) |
| Yes | 53 (8.0%) |
| **Worst CDG at discharge** |  |
| None | 611 (92%) |
| 1 | 14 (2.1%) |
| 2 | 23 (3.5%) |
| 3a | 1 (0.2%) |
| 3b | 9 (1.4%) |
| 4 | 2 (0.3%) |
| 5 | 4 (0.6%) |
| **Which AE at discharge** |  |
| None | 611 (92%) |
| New neurological deficit   - 12 classified as CDG 1 - 1 classified as CDG 2 (dexamethasone administration | 13 (2.0%) |
| Miscellaneous | 13 (2.0%) |
| Urinary tract infection | 5 (0.8%) |
| Pulmonary embolism | 5 (0.8%) |
| Postoperative hemorrhage | 4 (0.6%) |
| Pneumonia | 4 (0.6%) |
| Death | 3 (0.5%) |
| Epileptic seizure | 2 (0.3%) |
| Cerebral infarction | 2 (0.3%) |
| Thrombosis | 2 (0.3%) |

**Online resource 3 AE characteristics**. Statistics presented: n (%). AE = adverse events, CDG = Clavien-Dindo classification grading (see Online resource 1), N = number

| **Characteristic** | **OR** | **95% CI** | **p-value** |
| --- | --- | --- | --- |
| **Age** | 1.01 | 0.98, 1.03 | 0.55 |
| **Sex** |  |  | 0.63 |
| Female | — | — |  |
| Male | 1.15 | 0.65, 2.07 |  |
| **ASA** | 1.60 | 1.01, 2.54 | **0.044** |
| **Primary surgery** |  |  | 0.19 |
| Yes | — | — |  |
| No | 1.59 | 0.79, 3.03 |  |
| **Number of craniotomies** |  |  | 0.26 |
| 1 | — | — |  |
| >1 | 2.02 | 0.74, 4.73 |  |
| Biopsy | 0.49 | 0.03, 2.40 |  |
| **Neocortical location** |  |  | **0.005** |
| Cortical | — | — |  |
| Cerebellar | 3.44 | 1.73, 6.95 |  |
| Deep | 1.72 | 0.76, 3.78 |  |
| Extraaxial | 2.86 | 0.63, 9.43 |  |
| **Central region** |  |  | 0.57 |
| No | — | — |  |
| Yes | 1.29 | 0.51, 2.80 |  |
| **Sidedness** |  |  | **0.037** |
| Left | — | — |  |
| Midline | 3.94 | 1.04, 12.3 |  |
| Right | 0.83 | 0.44, 1.57 |  |
| Both | 2.78 | 0.87, 7.60 |  |
| **Urgency of the operation** |  |  | 0.93 |
| Elective | — | — |  |
| Emergency | 1.04 | 0.38, 2.37 |  |

**
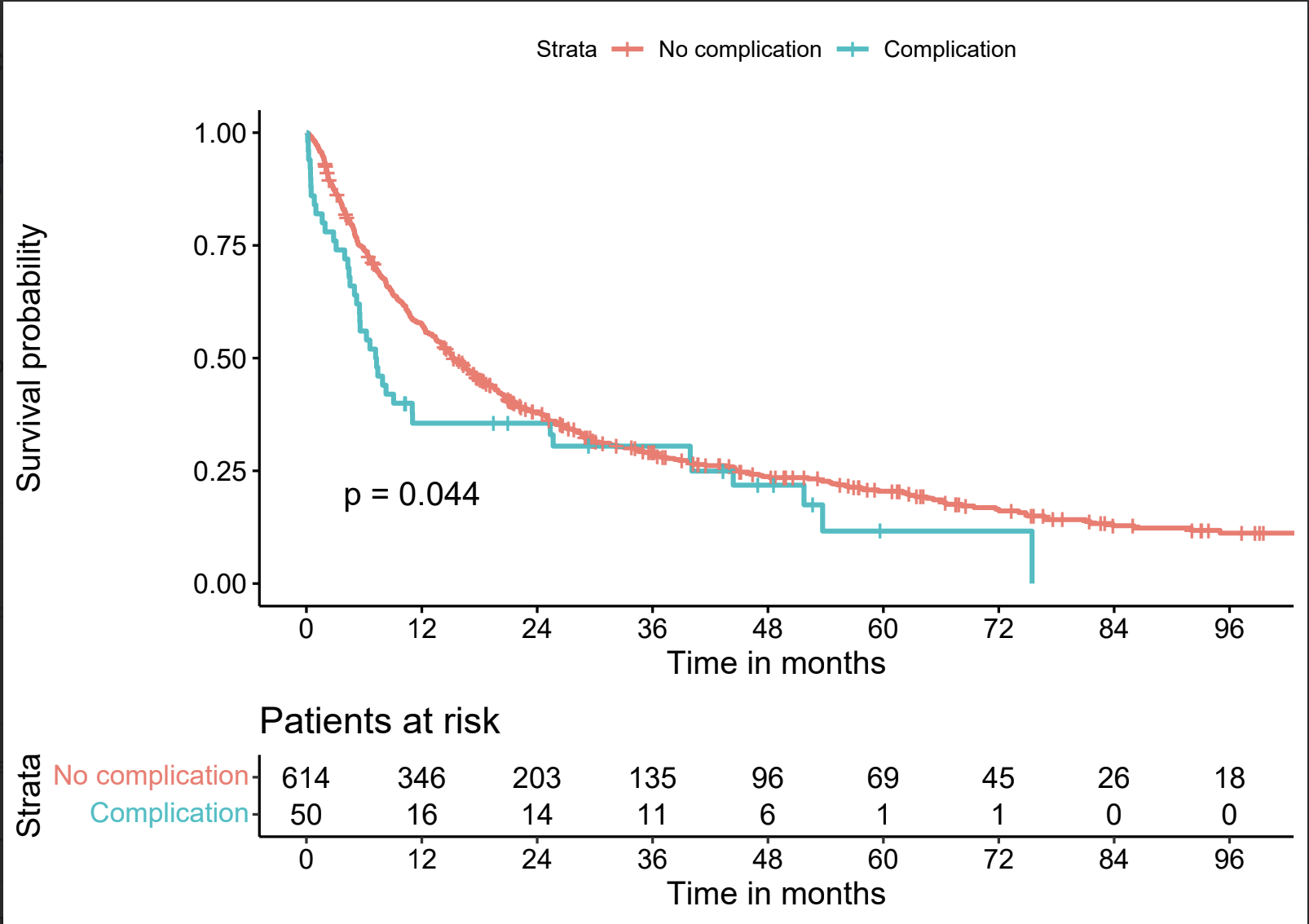
Online resource 4 Univariate logistic regression model of major AE at discharge.** Major AE at discharge were considered as CDG >= 2 or new neurological deficits. AE = adverse events, N = number, OR = Odds Ratio, CI = confidence interval, ASA = American Society of Anesthesiologists. Statistically significant p-values are marked in bold.

**Online resource 5 Overall survival stratified by major AE at discharge**. Kaplan-Meier curve with log-rank statistic of patients stratified by the occurrence of early postoperative AE. Major AE at discharge were considered as CDG ≥ 2 or new neurological deficits. AE = adverse events


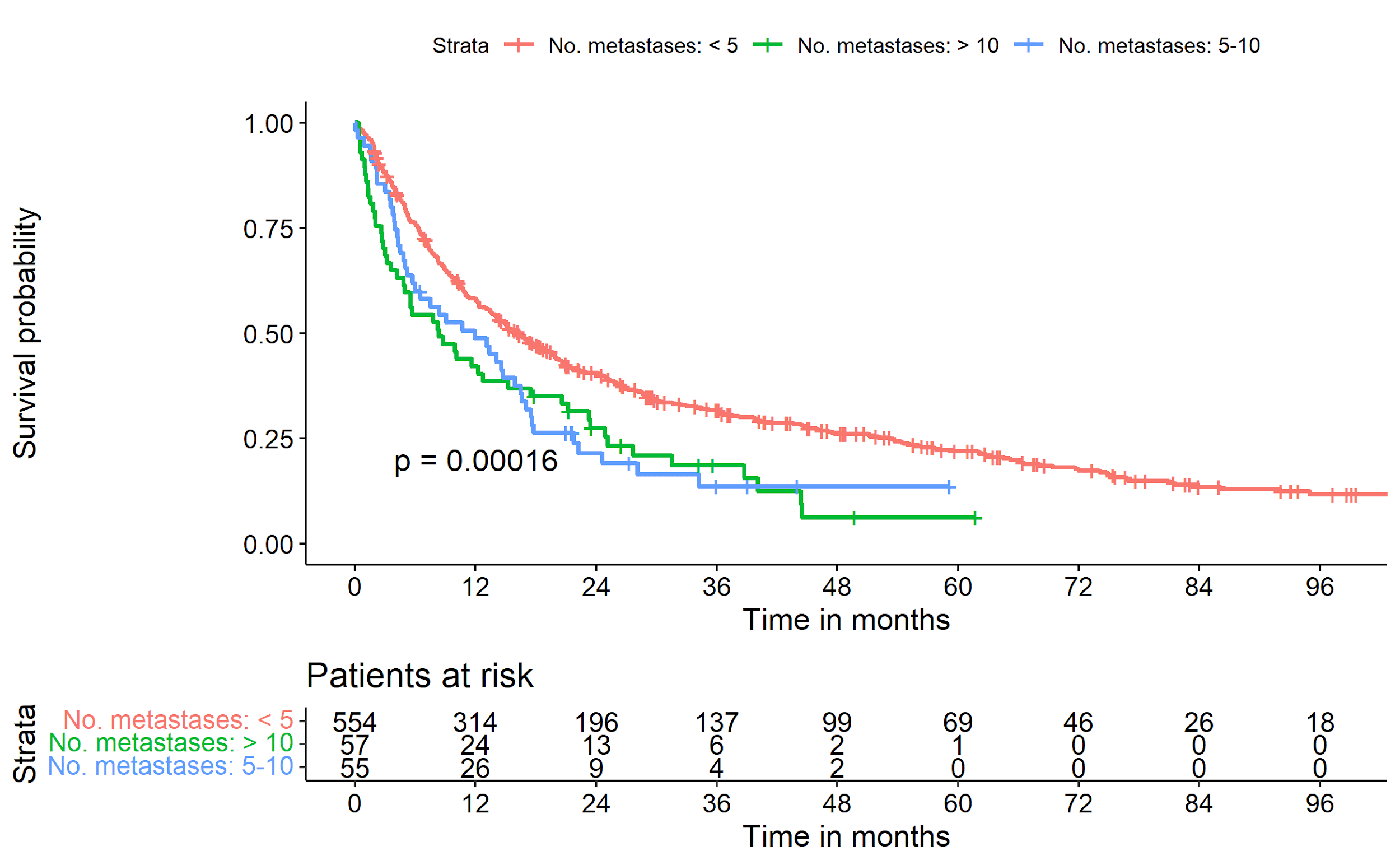


**Online resource 6 Overall survival stratified by number of BM**. Kaplan-Meier curve of patients stratified by the number of BM present at the timepoint of surgery, BM = brain metastases
